# Antiretroviral Treatment Adherence: Knowledge and Experiences among Adolescents and Young Adults in Soweto, South Africa

**DOI:** 10.1155/2017/5192516

**Published:** 2017-03-20

**Authors:** Stefanie Hornschuh, Janan Janine Dietrich, Celokuhle Tshabalala, Fatima Laher

**Affiliations:** Perinatal HIV Research Unit, Chris Hani Baragwanath Academic Hospital, Faculty of Health Sciences, University of the Witwatersrand, Gauteng 1864, South Africa

## Abstract

Human immunodeficiency virus (HIV) management of adolescents and young adults (AYAs) is particularly pertinent to sub-Saharan Africa, where the pediatric HIV burden is marked. Antiretroviral treatment (ART) adherence is a major challenge for AYAs. This qualitative study explored knowledge and experiences of adherence amongst AYAs attending treatment at the Perinatal HIV Research Unit (PHRU), Soweto, South Africa. Four focus group discussions (FGDs) and eight in-depth interviews (IDIs) were conducted with HIV-infected 15–25-year-old ART recipients. Transcripts were coded thematically. Participants (*n* = 26) were aged median 18.5 years, 59.1% female and 69.2% virally suppressed <400 cp/ml. Three main themes emerged during FGDs and IDIs: (i) correct knowledge about how to be adherent, benefits, and nonadherence consequences, (ii) social, personal, and medication-related barriers to adherence, and (iii) reminder, concealment, and motivational strategies to optimize adherence. Interventions to improve AYA adherence could focus on practical strategies, including status disclosure and medication concealment.

## 1. Introduction

The highest global burden of HIV is in South Africa, where prevalence was 12.2% (6.4 million people) in 2012 [1]. Amongst AYAs aged 15 to 24 years, prevalence was 7.1% [1]. About two-thirds of infected AYAs in South Africa acquired the virus through vertical transmission [2]. Before South Africa implemented prevention of mother-to-child transmission interventions, an estimated 60.000 perinatal HIV infections occurred per annum [3]; since then, improved ART access has reduced this number drastically. In 2013 alone, there were still 16.000 new infections in the 0–14-year-old age group in South Africa [4]. With ART advances, these children can and do survive into adolescence and adulthood [5, 6].

ART use results in substantial reductions in HIV-related morbidity and mortality [7, 8]. However, for ART to be effective, sustained viral suppression must be achieved through near-perfect pill adherence [9–11]. Lifelong adherence to daily pills is a known challenge for patients [12]. Nonadherence may result in drug resistance, treatment failure, and subsequent reduced treatment options [13, 14].

A recent study conducted in Zimbabwe between 2004 and 2010 to examine whether adolescents treated for HIV achieved similar treatment outcomes to adults found that HIV-infected adolescents are at higher risk for treatment failure than their adult counterparts [15]. Therefore, knowledge about adherence is critical, and there is a body of publications about adherence barriers and facilitators in the southern African context, especially quantitative research amongst adult patients [16]. Amongst adult patients in the region, various barriers to ART adherence have been identified: fear of HIV status disclosure, HIV-related stigma, alcohol use and drug abuse, forgetfulness, complicated ART regimens, pill burden, side effects, transportation costs, and financial constraints [17, 18]. In one study in South Africa, Peltzer et al. [19] found that determinants for adult nonadherence to ART included poor environment, experiences of HIV-related discrimination, and use of herbal medicine. Determinants of ART adherence included low depression scores, good adherence information, behavioural skills, and social support [19].

It is possible that many of the adherence barriers and facilitators for adults also apply to AYAs, but the latter's higher risk treatment failure does seem to suggest that additional concerns may need to be considered if we are to understand AYA adherence. AYAs in general are particularly vulnerable to adherence problems as they experience unique obstacles owing to the changes during adolescence, including psychological, cognitive, and social development [20, 21]. Even so, there are fewer publications focusing on AYA adherence in Africa. One qualitative study with 23 AYAs in Kenya found that adherence was affected by the need for secrecy to avoid inadvertent disclosure and stigmatisation, pill burden, medication fatigue, lack of planning when travelling, and using adherence as leverage in conflicts [22]. Our study qualitatively explored the experiences of HIV-infected AYAs with regard to ARV adherence.

## 2. Methods

We conducted a qualitative study through FGDs, IDIs, and a demographics questionnaire.

### 2.1. Setting

The study was conducted from March to August 2012 at the PHRU, located at the Chris Hani Baragwanath Academic Hospital in Soweto, South Africa. Soweto is a group of urban townships southwest of Johannesburg, with an estimated population of over one million people [23]. PHRU offers HIV testing, prevention, and treatment for infants, children, adolescents, and adults. At PHRU, HIV-infected AYAs were treated at either its pediatric or adult HIV clinic.

### 2.2. Participants

Eligibility criteria were age between 15 and 25 years at the time of study, HIV infection by self-report to study staff, receipt of ART at either the PHRU adult or pediatric treatment clinic, and ability to provide voluntary written informed consent and, if applicable, assent.

### 2.3. Data Collection Procedures

Potential participants were identified via the PHRU clinic database matching study age and treatment criteria. Potential participants, and their parents for those below 18 years of age, were briefly told about the study telephonically by a study representative and invited to a private room at the PHRU to receive information in full. All participants who provided voluntary written informed consent, and if applicable assent, were assisted by a female interviewer to complete a brief questionnaire about sociodemographic information. The questionnaire structure has been described previously and collected information about HIV transmission mode, HIV status disclosure, and socioeconomic status [24].

Four FGDs were first conducted to identify major themes. Once these were completed, AYAs who had not participated in the FGDs were invited to the IDIs, which attempted to elicit, in an environment without the interference of peer influence, more detailed information on the main themes which had been identified. All interviews were organized in the same location nearby to but removed from the clinic to create similar conditions.

#### 2.3.1. Focus Group Discussions

The semistructured FGD interview guide questions elicited the level of knowledge and participant perceptions about adherence and adherence barriers and facilitators.

FGD questions about ART adherence were as follows:

1. Tell me everything you know about ART adherence.
2. Who explained to you how to be adherent?
3. Everyone who takes medication every day has days when they feel like they don't want to take medication. Who can tell us what happens to them on those days?
4. Can you think of reasons why young people can't take their medication as they are supposed to do?

IDI questions about ART adherence were as follows:

1. FGD questions listed above were also asked in IDIs.
2. In your opinion, why is it important for someone who is HIV positive to take his/her medication properly?
3. Did you ever face problems with your medication (e.g., side effects, switch of medication)? What were your experiences?

The interview guide was piloted with a convenience sample of three voluntary AYA patients from the pediatric HIV clinic who gave their opinions regarding the questions and their understanding so that the guide could be refined.

With the finalized interview guide, four FGDs were conducted, each consisting of three to six participants. The FGDs were stratified by clinic and gender: (1) female patients from the pediatric clinic, (2) female patients from the adult clinic, (3) male patients from the pediatric clinic, and (4) a mixed group of male and female patients from the adult clinic. FGDs were between 45 and 90 minutes' long and were audio-recorded. Interviews were facilitated by two young local multilingual female interviewers who were experienced in AYA qualitative research and trained on HIV and treatment issues for AYA. Questions were open-ended and probed where necessary to ensure robust data generation. The FGDs were conducted in a mix of English, Zulu, and Sotho [local languages in South Africa], with the facilitators being fluent in all three languages. Audio-recorded transcripts were transcribed and translated into English.

#### 2.3.2. In-Depth Interviews

Eight IDIs were conducted, each around 60 minutes long and audio-recorded. IDI participants were not the same as the FGD participants. Interviews were facilitated by one of the two female interviewers who had conducted the FGDs. The question guide asked the same questions that had been in the FGDs to confirm whether responses that had been given in a peer-context would be different from those given without peer influence, and extra questions probed the level of understanding about the importance of adherence and the experiences of adverse effects of medications (see FGD and IDI questions about antiretroviral therapy adherence in Section 2.3.1). Audio-recorded transcripts were transcribed and translated into English.

#### 2.3.3. Biological Marker of Adherence

Because ART adherence is a behaviour that can be linked with a biological marker, we collated the most recent HIV-1 viral load of the participants from the clinic records. However, viral load data was unknown to the interviewers at the time of the interviews with the participants so as to prevent influencing the course of the discussion.

### 2.4. Ethical and Community Approval

Ethical approval was obtained from the University of the Witwatersrand Human Research Ethics Committee. The Soweto adolescent community advisory board found that the study was relevant and appropriate. Study participant confidentiality was maintained by use of study numbers (not names) during the study proceedings and on the study documents. Participants, and parents/legal guardians if applicable, were given a reimbursement of R50 (~USD4) for transport costs.

### 2.5. Data Analysis

Quantitative data gained from the sociodemographic questionnaire were entered into Microsoft Office Excel to ensure accuracy across the data. Descriptive statistics and frequencies were calculated using Statistical Package for Social Sciences (SPSS) version 22.0. The most recent HIV-1 viral load was used to determine participants overall adherence; participants that were virally suppressed <400 cp/ml versus those that were not virally suppressed >400 cp/ml.

The FGD and IDI transcripts were hand-coded by the primary author using thematic analysis. First, the primary author entered a process of data immersion by reading and rereading transcripts. Thereafter, the primary investigator developed a code-book with the second author. Codes were developed based on the interview guide and addressed the following aspects: correct knowledge about how to be adherent; correct knowledge about adherence benefits and nonadherence consequences; social, personal, and medication-related barriers to adherence; and reminder, concealment, and motivational strategies to optimize adherence. Codes were then grouped into categories to develop subthemes. Relevant quotations selected from the transcripts were used to support the identified themes.

## 3. Results

### 3.1. Participant Characteristics

Overall, 186 ART patients between the ages of 15 and 25 years were identified; 123 had documented telephone numbers in the clinic databases or patient records and 26 participated in the study. The response rate for study participation was 22.4% (17/76) for females and 19.1% (9/47) for males. A total of 18 participants participated in the FGDs, 13 females (72.2%) and 5 males (22.8%). IDIs were conducted with 8 participants, equally divided by gender as 4 females and 4 males.

Table 1 shows participant characteristics. The median age of participants was 18.5 years [interquartile range (IQR) 17.0–21.5 years]. Seventeen (65.4%) participants indicated that they had acquired HIV through vertical transmission. The majority (*n* = 17, 65.4%) were receiving ART at the pediatric clinic facility. The HIV status of all participants was known to at least one household member, most commonly their siblings (*n* = 14, 53.8%), biological mother (*n* = 12, 46.2%), and grandparents (*n* = 12, 46.2%). Sixteen (61.5%) participants were classified as food insecure. The most recent viral load laboratory results showed that 18/26 (69.2%) participants were virally suppressed (<400 cp/ml) at the time of participation.

**Figure 1:**
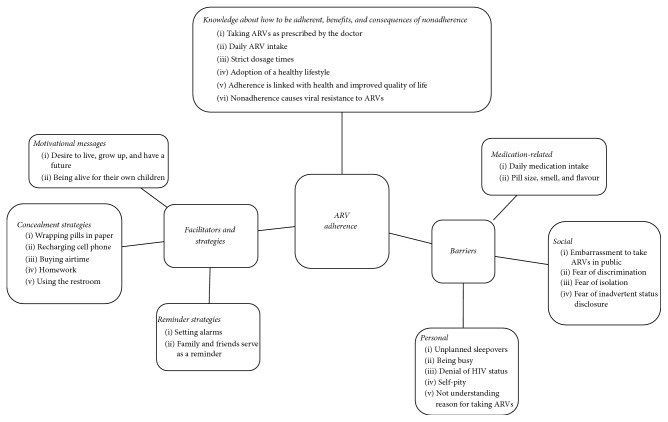
Summary of themes and subthemes emerging from FGDs and IDIs.

Three main themes emerged during FGDs and IDIs: (i) AYAs have correct knowledge about how to be adherent, benefits of adherence, and the consequences of nonadherence, (ii) despite correct theoretical knowledge about adherence, real life barriers existed that could result in suboptimal adherence, and (iii) there were facilitators and strategies to optimize adherence (Figure 1).

### 3.2. Correct Knowledge about How to Be Adherent, Benefits, and Consequences of Nonadherence

Most participants provided accurate basic knowledge about how to take their antiretrovirals (ARVs) properly. Most FGD participants agreed that the medication schedule should be followed as prescribed. FGD and IDI participants emphasized that taking medication every day without missing days and that following strict dosing times were important parts of adherence. However, though correct information about dosing was known to participants, the rationale for the dosing schedule was not always known. A 25-year-old female FGD participant said “If they [the doctors] say 3 times a day and you take 2, they have their reasons why you have to take it 3 times a day.” Two participants agreed that a time period of twelve hours should lapse between the first and the second doses. Two participants emphasized that adopting a healthy lifestyle was also part of adherence to treatment: “You should start making decisions about [your] life, like for instance eating healthy food and living a healthy lifestyle” (17-year-old female FGD participant).

Many said they had started treatment in childhood. Explanations about how and why treatment should be taken were often introduced by the clinic staff, but three IDI participants said a family member had taught them. One 22-year-old female IDI participant, infected through vertical transmission, said that “both the doctor and my aunt explained perfectly well to me and the importance of me taking it [ARVs].”

Participants stated their perceptions about the benefits of ARV adherence. Three participants agreed that outcomes of strict adherence to ARVs would be prolonged life and improvement of quality of life. An 18-year-old male IDI participant stated that strict adherence would decrease the risk of HIV transmission.

Even though participants had difficulties in explaining the biological rationale of why ARVs had to be taken as prescribed, they were aware that there were consequences of nonadherence. They showed awareness that nonadherence could cause viral resistance to ARVs. For instance, a 19-year-old female FGD participant stated “If they [HIV patients] won't be taking their medication they'll end up getting different kind of viruses and that will influence them…” Another participant stated “They [doctors] told me that if I miss a dose the virus will grow and end up showing itself” (17-year-old male FGD participant).

Others perceived suboptimal ARV adherence to decrease overall health and quality of life, as expressed by one 21-year-old male IDI participant: “It is important [being adherent to ARVs] because as you are seeing me today as strong as I am, it is because of those pills and if you don't take them you go back to getting sick.” A female FGD participant, aged 24 years, talked of the outcome of her experience being nonadherent to her ARVs as such “You'll pick up sickness, be in hospital in and out….”

### 3.3. Barriers to Suboptimal Adherence

At first, participants were uncomfortable admitting that they missed doses. Those that said they had been nonadherent declared to have done so unintentionally. Participants became more comfortable describing possible reasons for suboptimal adherence when the interviewer asked them to talk about “others” and not themselves necessarily. In this context of referring to others, participants easily volunteered personal, social, and medication-related reasons for suboptimal adherence and eased into talking about their own barriers to adherence.

#### 3.3.1. Personal Barriers

Some participants said that when they became forgetful about their medication reminder system, their adherence would suffer. One 18-year-old female FGD participant said “Sometimes I cannot carry a pill reminder with me to school because the last time I did that it got lost at school and it cost me to buy another one.” Five other participants admitted that they forgot taking their medication during an unplanned sleepover or simply forgot to take a dose while being in a rush or being busy. A 16-year-old male IDI participant described such a situation during a school trip: “I was not at home and I was on a school trip…I forgot them [ARVs], because I was too excited about the trip.”

Another personal barrier to adherence was a level of denial and anger about childhood HIV persisting into adolescence and young adulthood despite treatment adherence in childhood. A 21-year-old female FGD participant, infected through vertical transmission, talked about how she used nonadherence to test out her disbelief that she would still have HIV after a whole childhood of taking treatment. “I am a very suicidal person…there are times where I would just give up…you are taking treatment…I'm still getting sick…I am still losing weight…but growing up realizing that this [ARVs] is something I [have] been drinking since I was a child – no man let me just live it like that [without ARVs]. Well I have been testing. I cannot say I have not been missing my doses – I have missed doses; I've defaulted a lot of times… I wanted to see what [was] going to happen to me and you find two to three months you are healthy and the next month you are out and you starting to get sick then you ask yourself why am I still sick with the very same sickness that I have been taking treatment for. Like, there [are] so many questions…”.

The need for assimilation and not wanting to feel different was another personal reason named for neglecting doses. A female FGD participant aged 17-years, infected through vertical transmission, expressed “… If there is a family gathering…and they are not taking them and I have to take them, I feel different and think why I should drink them.”

There was also mention of nonadherence as a means of dealing with conflict situations, with the intention to do self-harm: “Me and my granny were not in good terms…I would do that [not taking ARVs] just to make a revenge because I would think she doesn't love me and it's better if I kill myself and not taking the pills” (18-year-old male IDI participant).

#### 3.3.2. Social Barriers

Social reasons for suboptimal adherence were heavily clustered around the idea of stigma. AYAs felt that taking medication in front of friends and family members would cause them to become embarrassed about having HIV, and furthermore they feared they would be discriminated against. Therefore, they generally took medication in secret so they could avoid inadvertent HIV status disclosure.

However, beyond stigma, there was also the idea that adherence could interfere with perceived normative AYA social behaviour. Drinking alcohol at parties was perceived as normative social behaviour, and it was perceived that ART and alcohol should not be taken together. One 24-year-old female FGD participant said “You are at a party and you are thinking about your medication so you thinking of going home fast. And what [is] bad is that you [are] not supposed to mix the medication with alcohol. So those are the challenges that we are facing as young people.”

#### 3.3.3. Medication-Related Barriers

Many participants expressed feeling tired or bored of taking their ARVs daily. A 24-year-old female FGD participant said “This thing of them [clinic staff] telling us to drink them [ARVs] every day and every day… it [is] just too much work. At least if other pills they make them to be syrups instead of pills.” Others reported being tired of the big size, smell, and taste of the pills. A 16-year-old male FGD participant explained that “the pills need a flavour and …taste…sometimes I feel like I don't want to take them anymore and…some of them are just so big.”

### 3.4. Facilitators and Strategies for Optimal Adherence

Participants described several strategies that they acquired to support optimal adherence to ARVs including reminder, concealment strategies, and motivational messages.

#### 3.4.1. Reminder Strategies

Participants mentioned different reminder methods including alarms, medication charts, family members, and friends. Four participants reported that they set daily alarms that reminded them to take their ARVs in time. However, for one female participant, setting alarms was risky, as she thought it could reveal her HIV status inadvertently to others. The best option for her was to disclose to a trusted person who would be able to remind her to take her daily ARVs.

Some participants stated they used their closest family and friends, who knew their status, as an additional reminder to take medication. A female FGD participant aged 16 years explained that “for me, drinking the medication in front of other people is not a problem. When me and my mother are visiting, my mother will tell me straight that I go and take my medication. Even if they ask me what the medication is for I'll tell them straight…and they keep quiet.”

#### 3.4.2. Concealment Strategies

In contrast, some participants were uncomfortable disclosing their HIV status to family members or friends, particularly when there was not enough trust. They acquired concealment strategies to take their ARVs in order to avoid inadvertent HIV status disclosure, discrimination, and isolation. Some participants reported wrapping their pills in paper or tissue to hide them from other people. A 25-year-old female participant said “The pills are always in my jean pockets or my breast inside the tissue. If I do not have water I opt for a chewing gum or some sweets. I put them one by one so that they won't ask any questions.” Others invented reasons to be able to take their ARVs secretly, such as needing to get away to recharge cell phones, buy airtime for cell phones, do homework, or go to the restroom.

#### 3.4.3. Motivational Messages

Many participants developed motivational messages with the desire to live, to grow up, to have a future and career. Those motivational messages kept them focused on being adherent to their ARVs despite the side effects that some of them experienced. For instance, one male IDI participant aged 20 years said “…I didn't want to become a statistics being another number…I wanted to live and continue my life and that's the reason why I am here, so I might as well live it.” Two others confessed that their main motivation for taking their ARVs properly is their child: “Now I have a reason why I should take my treatment, I have a child now, so I have to look up, that you know that not only for me, I am not living for myself now, this I [have] somebody to look out for…” (21-year-old FGD female participant).

The majority of participants stated that they accepted their HIV status and were driven to be adherent by the desire to gain a long and healthy future life: “Why should I be ashamed of my life, this is how I have to live my life, take my pills, because without them I could get AIDS and AIDS kills…If I get ashamed of them [ARVs] it means I am ashamed of myself” (16-year-old male FGD participant).

## 4. Discussion

This qualitative study is one of the few studies to explore the knowledge in regard to adherence barriers and facilitators and experiences of ART adherence with AYAs who were mostly perinatally infected and from a developing setting.

As expected, AYAs in our study shared many adherence challenges previously identified in quantitative research amongst adult patients: fear of HIV status disclosure, HIV-related stigma, alcohol use, forgetfulness, and depression [17–19]. However, not all challenges were mutual. Our AYAs did not emphasize ART side effects as a challenge, even though studies in adults have found this. And unlike adults [19], AYAs did not speak of financial constraints. This is understandable because, even though most come from impoverished circumstances, AYAs may not hold financial responsibility in the household. A study in the South African private sector, which serves better-resourced patients who are able to pay for care, found 43.6%–55.8% viral suppression at 24 months on treatment in 10–30-year-olds, and correspondingly poor adherence [25]. In contrast, almost seventy percent of our study participants had suppressed viral loads, admittedly a biased sample because their willingness to participate in the study may reflect better adherence behaviours, but it is similar to AYAs in other resource-limited settings [26]. Taken together, it demonstrates that AYAs can overcome poor environments to maintain excellent treatment outcomes. The self-motivating strategies for adherence described by AYAs in our study show their resilience in trying to achieve this.

Our study identified unique adherence challenges that AYAs face, which are different from adults; some of these may be unique to AYAs who were infected at a young age. For example, we described a perinatally infected AYA who doubted persistence of the virus after a childhood of ART adherence and used nonadherence as a method to establish the ongoing pathogenicity of the disease.

Another issue for the subset of AYAs who were infected at a very young age is status disclosure and how that could affect adherence. In our study, AYAs, even perinatally infected ones, demonstrated correct basic knowledge about medication adherence and its benefits as well as consequences of nonadherence. Indeed, many have been in care since childhood and credit their knowledge source to their healthcare providers. This is encouraging because it implies that, for the perinatally infected AYAs in this study, healthcare providers have generally been able to transfer knowledge from parents and caregivers to the patients as they grow into adolescence. Clinical care that includes counselling, a supportive environment, a relationship of trust with the provider, and open discussion of patient concerns can help patients develop adequate adherence strategies [27–29]. This kind of optimal care is facilitated when vertically infected children are made aware of their HIV diagnosis in a timely manner [30]. Many children in developing countries are not told about their HIV diagnosis [31–33]. In a recent study conducted among pediatric ART children aged 4 to 17 years in Gauteng, South Africa, only 34% were disclosed about their HIV status [32]. Indeed, it is plausible that some of the one-fifth of adolescents in our study who stated that they do not know their infection route are perinatal infections who have not been disclosed to, placing them at risk of not understanding the importance of adherence, and dropping out of adherence, as they become adults. The disclosure process and the timing of disclosure as well as the relationship with the healthcare provider and their parents play an important role in regard to ARV medication management for AYA patients and are an area that requires further research [33, 34]. Learning about HIV status in a timely and appropriate manner has benefits in achieving long-term disease management and improvement of ARV adherence [18, 30].

Other challenges for AYA stem from how ART interrelates with the normative increased socialisation of adolescence. AYA described adherence challenges in relation to taking alcohol with medication. But mostly, they feared that public pill taking would inadvertently disclose their status, embarrass them, and stigmatise them at sleepovers, parties, family functions, and hanging out with friends. As with adults, this was a prominent reason for skipping doses in our study and supported by findings from another study conducted in Chicago, United States, among adolescents and young adults [28]. Although the majority claimed that at least one household member knew their status, few to very close friends were aware of their status, and this confirms assertions that the fear of stigma remains high even in high HIV prevalence settings [22, 35].

*Limitations.* Because of the poor participation and the fairly high proportion of virally suppressed participants in our sample, we do not know to what extent these findings would be valid for participants who have very poor adherence and are lost to follow-up. A possible reason for the poor participation could be fear of implied HIV status disclosure in a peer group setting.

The presence of other peers during FGDs could have affected participants' responses, but we conducted IDIs with different individuals to ensure that data was not only received from a peer-influenced environment. Although there was evidence of social desirability bias, we attempted to limit it by encouraging participants to talk about nonadherence in others when they did not want to admit to it initially in themselves. This gave way to personal anecdotes of times they had experienced nonadherence.

## 5. Conclusion

Despite having good knowledge about ART, AYAs may face circumstances which can derail optimal adherence. AYAs may present with specific adherence problems, such as denial about HIV persistence after a childhood of treatment and the issues that HIV/AIDS poses to them during a phase of development in which social assimilation gains prominence in their lives. AYAs may find it difficult to open up about these circumstances as they struggle internally to resolve them. It may be useful for the healthcare practitioner facing a poorly adherent AYA patient to learn from the strategy we employed in our interviews: inviting AYAs to share general, not necessarily personal, barriers to adherence. Regardless, in a world where HIV discrimination remains a reality, secretiveness may be a practical and palatable strategy which a healthcare worker can suggest to AYAs to help maintain adherence while public disclosure is unthinkable for the individual. Parallel interventions may be motivational messaging focusing on the known long-term health, longevity, and prevention benefits of achieving viral suppression through adherence and practical strategies for status disclosure where appropriate.

## Figures and Tables

**Table 1:** Characteristics of adolescents and young adults who participated in FGDs and IDIs (*n* = 26).

Characteristics	
Median age, years (IQR)	18.5 (17.0–21.5)
Gender, *n* (%)	
Female	17 (59.1)
Male	9 (40.9)
Self-reported mode of HIV acquisition	
Vertical transmission	17 (65.4)
Heterosexual transmission	4 (15.4)
Unknown	5 (19.2)
Treatment clinic attendance, *n* (%)	
Adult clinic	9 (34.6)
Pediatric clinic	17 (65.4)
People to whom HIV status is disclosed^*∗*^, *n* (%)	
Biological mother	12 (46.2)
Biological father	2 (7.7)
Sibling/s	14 (53.8)
Grandparent/s	12 (46.2)
Cousin/s	4 (15.4)
Partner/s	4 (15.4)
Friend/s	3 (11.8)
Teacher/s	1 (3.8)
Other	8 (30.8)
Food security, *n* (%)	
Insecure	16 (61.5)
Marginally secure	5 (19.2)
Secure	5 (19.2)

Biological marker of ART adherence	
Virologically suppressed < 400 cp/ml, *n* (%)	18 (69.2)
Female^*∗∗*^	12/17 (70.6)
Male	6/9 (66.7)

^*∗*^Multiple responses allowed.

^*∗∗*^One missing value for most recent viral load.
